# Characteristics and Geographic Variability of United States Assisted Living Communities by Chain Status

**DOI:** 10.1093/geroni/igaf122.1406

**Published:** 2025-12-31

**Authors:** Cassandra Hua, Leya Kurzhiparambil, Lindsey Smith, Gauri Gadkari, John Bowblis, Momotazur Rahman, Kali Thomas

**Affiliations:** University of Massachusetts Lowell, Lowell, Massachusetts, United States; University of Massachusetts Lowell, Lowell, Massachusetts, United States; Oregon Health & Science University, Portland, Oregon, United States; Brown University, Providence, Rhode Island, United States; Miami University, Oxford, Ohio, United States; Brown University, Providence, Rhode Island, United States; Johns Hopkins University, Baltimore, Maryland, United States

## Abstract

Previous studies indicate chains operate nursing homes with wealthier, higher-acuity residents, though state policies like Medicaid generosity create variation. We analyzed the distribution and characteristics of chain-affiliated versus non-chain ALs. We connected AL chain status (2021 data from National Investment Center, Argentum, and Dun & Bradstreet) to community characteristics (American Community Survey) and AL resident characteristics (Medicare enrollment and claims). In state-level analysis, we compared the number of chain vs. nonchain ALs per 100,000 adults aged 65+ using a scatterplot and Pearson’s r. In AL-level analysis, we compared characteristics by chain status using t-tests and chi-squared tests. Our sample included ALs with a capacity of 25+ beds with at least 10 fee-for-service Medicare beneficiaries, including 6,409 ALs across the 48 contiguous U.S. states. We found a modest correlation between the number of chain and nonchain AL beds per 100,000 older adults in each state (r = 0.29, p = 0.047). Some states, including Utah, Indiana, and Idaho, were “chain dominant.” Utah, for example, had 1,480 chain operated AL beds per 100,000 older adults compared to just 16 nonchain beds. Residents in chain-owned ALs were more likely to have dementia than those in nonchain ALs (36% vs. 30%, p < 0.001). Significantly more residents in nonchain ALs were dually eligible for Medicare and Medicaid (40% vs. 21%, p < 0.001). County characteristics (e.g., median income) did not differ significantly by chain status. Findings suggest that, similar to nursing homes, chains may serve residents with greater care needs and less financial disadvantage.

